# Evaluation of Thermal Properties of 3D Spacer Technical Materials in Cold Environments using 3D Printing Technology

**DOI:** 10.3390/polym11091438

**Published:** 2019-09-02

**Authors:** Ran-i Eom, Hyojeong Lee, Yejin Lee

**Affiliations:** 1 Research Institute of Human Ecology, Chungnam National University, Yuseong, Daejeon 34134, Korea; 2 Department of Fashion Design & Merchandising, Kongju National University, Gongju, Chungcheongnam-do 32588, Korea; 3 Department of Clothing & Textiles, Chungnam National University, Yuseong, Daejeon 34134, Korea

**Keywords:** technical material, 3D spacer fabric, heat transfer, heat insulation, 3D printing technology

## Abstract

Novel materials have been recently developed for coping with various environmental factors. Generally, to improve the thermal comfort to humans in cold environments, securing an air layer is important. Therefore, this study analyzed the thermal properties of 3D spacer technical materials, 3D printed using thermoplastic polyurethane, according to the structural changes. Four 3D spacer technical material structures were designed with varying pore size and thickness. These samples were moved into a cold climate chamber (temperature 5 ± 1 °C, relative humidity (60 ± 5)%, wind velocity ≤0.2 m/s) and placed on a heating plate set to 30 °C. The surface and internal temperatures were measured after 0, 10, 20, and 30 min and then 10 min after turning off the heating plate. When heat was continuously supplied, the 3D spacer technical material with large pores and a thick air layer showed superior insulation among the materials. However, when no heat was supplied, the air gap thickness dominantly affected thermal insulation, regardless of the pore size. Hence, increasing the air gap is more beneficial than increasing the pore size. Notably, we found that the air gap can increase insulation efficiency, which is of importance to the new concept of 3D printing an interlining.

## 1. Introduction

The environment is constantly changing due to global warming and increasing fine dust levels, which has necessitated emphasis on the function of clothing to protect the body. Increasingly high summer temperatures and low winter temperatures are of concern worldwide. Against these environmental transitions, clothing provides comfort from heat and cold by serving as a protective barrier between the human body and the environment [1]. The extent of heat and cold experienced by the human body are affected by environmental factors, including air temperature, radiation temperature, humidity, and air currents, as well as physiological factors, including amount of clothing and amount of activity. The thermal comfort provided by clothing is affected at the scales of the textile, thread, fabric, and clothing [2,3,4,5,6]. Furthermore, cloth-scale effects depend on the shape of the clothing, clothing pressure, area of body exposure, and method of body ventilation [7,8,9,10]. Heat transfer through clothing is affected by the convective current between clothing and environment, conduction, and radiation [11,12]. Previous studies pertaining to the heat transfer of clothing include one by Rhie [13], who determined the thermal resistivity between the clothing, body, and environment by modeling sleeves and investigated how the fabric and air gap thickness affect thermal transmission. In another study, Das et al. [14] developed a mathematical model to predict thermal transmission for multilayer structures with air between two fabrics. Moreover, Shen et al. [15] developed a heterogeneous fabric model for calculating the contact heat transfer coefficients for yarn.

The most important feature for clothing suited for low-temperature environments is insulation. To enhance insulation, heat conductivity must be low, and the material must be compact and airproof with fine pores and low ventilation. The air gap within clothing is of utmost importance to its insulating properties [16], so designing a material to maintain this air layer is very important. Heat loss and air gaps vary by body part. Psikuta et al. [17] stated that the back and waist may have more fixed air gaps than the abdomen due to the curvature of the human body. Furthermore, air gaps can be affected by the clothing design, production method, and physical properties of the fabric [8,9,10,18]. Chen et al. [18] and Weder et al. [19], showed that loose clothing has 30% more insulation than tight clothing. Song and Kwon [20], in their study of thermal properties for comfortable winter insulation clothing, demonstrated that clothing insulation is most related to the insulation of the fabric, and that the significant factors include the permeability, weight, and thickness of the fabric. 

Long-term exposure to cold environments reduces comfort and agility, thereby reducing the performance of the material and increasing the risk of injury. Therefore, insulating materials that actively utilize the sorption heat generation by clothing via the absorption of body moisture (ADVANSA Thermo°Cool^®^, a type of thermoregulation material), phase change materials (Outlast^®^, Jockey^®^), and smart materials that utilize microencapsulation and electronics are being developed. In addition, Wieme et al. [21] analyzed the effects of thermal conductivity by comparing polymer-based composite materials among various insulating materials. Duan [22] and Wondu [23] also confirmed that thermoplastic polyurethane (TPU) and polyurethane (PU) polymers can create conductive networks. Meanwhile, 3D spacer fabrics composed of two fabric layers with vertical pile yarns are being actively developed and studied. For example, Mao and Russell [24] studied the thermal insulation properties of spacer fabrics with a woolen fiber surface and Abounaim et al. [25] developed curvilinear 3D multilayer spacers that serve as thermoplastic composites to predict the mechanical performance. Chen et al. [26] analyzed the compression and impact behavior of 3D structured composites based on warp-knitted spacer fabrics. The composites obtained using spacer fabrics with a close outer layer structure, finer spacer yarn, larger spacer yarn inclination angle, and higher thickness were better with respect to impact performance owing to their superior energy absorption capabilities. 

However, despite the insulation-related merits of 3D spacer fabrics, most preceding studies have only evaluated the mechanical performance of these fabrics in terms of their flexibility. Studies on their insulation performance are lacking. In addition, conventional 3D spacer fabrics do not employ 3D printing technology to create fabrics. 

In one thermal conductivity study using a 3D spacer fabric, the fabric density had the greatest effect on the thermal properties of the spacer fabric [27]. However, this study only evaluated knitted 3D spacer fabrics. In another study [28], the thermal insulation of 3D spacer fabrics was analyzed according to thickness and density, but these materials were knitted from polyester and nylon. On the other hand, fabric model construction through 3D printing can be applied to the tetrahedral, pyramidal lattice, X-type, and three-dimensional Kagome lattice structures, based on 2D lattice structures [29], so the 3D printing of fabrics has the advantage of being able to produce variable modeling. In a recent previous study [30], 3D printing was used to print a polylactic acid (PLA) polymer onto a fabric surface, thereby attempting adhesion of the polymer and woven fabrics. The role of polymers that may be combined with clothing is becoming important. Now, the possibility of apparel materials utilizing 3D printing has been confirmed [31], and the design of flexible and elastic fabrics that maintain the strength of 3D printing results has been attempted [32]. It would now be meaningful to determine a thermal conductivity model for fabrics produced through 3D printing technology. Continuous efforts to develop new materials that will efficiently improve the heat retention capacity are essential. Therefore, this study analyzes the thermal transmission properties of 3D printed materials with 3D spacer fabric structures. In other words, thermal transmission properties are analyzed according to pore size and thickness for use in cold weather clothing.

## 2. Materials and Methods

### 2.1. 3D Modeling of Materials

**Figure 1:**
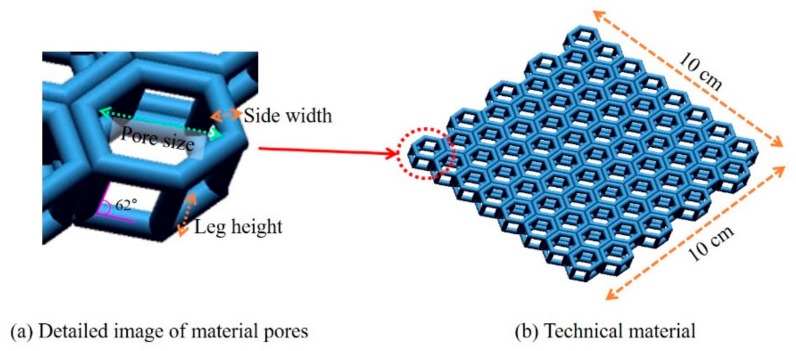
Technical material design according to different side widths, leg heights, and pore sizes.

The fabrics used in this study were 3D modeled by referencing commercial 3D spacer fabrics and produced through 3D printing. Thus, the 3D structure with spacer yarn between two 3D spacer fabrics [27] was reflected. The pore size and thickness were set as variables, as these are considered to be the main influencing factors in the evaluation of the physical properties of commercial 3D spacer fabrics [28]. The detailed structure of the designed technical material is shown in Figure 1a, with the top and bottom in hexagonal shape, composed of the “side width” and “pore size”, connected by the “leg height” to formulate an air gap between the two layers. The slope of the leg, which determines the thickness of the 3D spacer fabric, is set as 62°. This is because most commercially available 3D spacer fabrics are yarns that are inclined between the surface and the back side [28]; hence, a slope is introduced even when the spacer yarn is bent. Overall, the sample was a 10 cm × 10 cm square model (Figure 1b). Four types of technical materials (A, B, C, D) were modeled with the side width, leg height, and pore size as variables. Materials A and B had a side width of 0.2 cm and pore size of 0.9 cm; hence, the pore size was large due to the thin side width. However, A and B had different leg heights of 0.5 cm and 1.5 cm, respectively. Materials C and D had a side width of 0.5 cm and pore size of 0.6 cm; hence, their pore size was slightly smaller than that of A and B because the side width was thicker. Similarly, C had a leg height of 0.5 cm, and D had a leg height of 1.5 cm (Table 1). SolidWorks 2016 (Dassault Systems) was used for the 3D modeling.

### 2.2. 3D Printing of Materials

**Figure 2:**
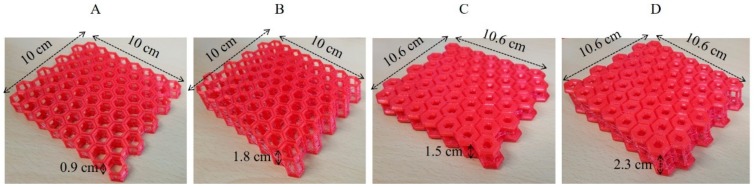
Four types of technical materials.

For the actual production of the four technical materials that were 3D modeled (A, B, C, D), the 3D modeling files were converted into files compatible with 3D printers by Cubicreator v3.6 (CUBICON Inc., Youngin, Korea). Next, 3D printing was carried out using the fused filament fabrication (FFF) method, with CUBICON Single Plus (CUBICON Inc., Korea). The flexible filament employed was TPU (HyVISION, Sungnam, Korea). Generally, TPU does not break well when bent, is easily restored to its original form, and does not stretch or tear [33]. Due to these properties, this is a printing material that is widely used in clothing manufacturing [34]. Therefore, TPU was chosen in this study to facilitate the combination of clothing with technical materials. The specifications of the TPU and printed technical materials are provided in Table 2 and Figure 2, respectively. At this time, the MFI (melt flow index) of the TPU used in the present study was higher than the MFI value of PLA (at 10 g/10 min) [35]. The four samples, on the other hand, were difficult to mechanically test on their own because they were the result of printing with a hexagonal design. Therefore, in this study, a tensile test was conducted to confirm the mechanical strength of the TPU material. The tensile properties of the TPU samples were measured according to ISO 527-2 [36]. The test piece had 75 mm gauge marks and tests were performed at a tensile velocity of 50 mm/min. The sample tensile strength was 11.7 MPa at 0.5 cm and 10.4 MPa at 1.0 cm, depending on the thickness. 

### 2.3. Temperature Measurement Analysis

**Figure 3:**
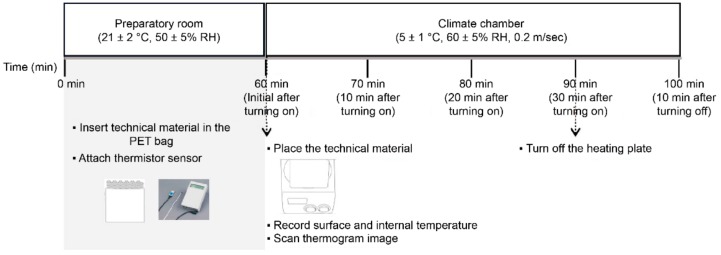
Experimental protocol for measuring the surface and internal temperatures of the technical materials.

Under the assumption that the four types of technical materials (A, B, C, D) would be inserted inside the clothing, pocket-shaped bags of 100% polyester fabric (yarn type: filament, construction: plain weaved, fabric type: taffeta, warp density: 41 cm, weft density: 33 cm, thickness: 0.07 mm, weight: 48.7 g/m^2^) were produced according to the technical materials’ size and height. The technical materials were located inside the pocket bags to measure the changes in the surface temperature and internal temperature. The experimental protocol is depicted in Figure 3.

**Figure 4:**
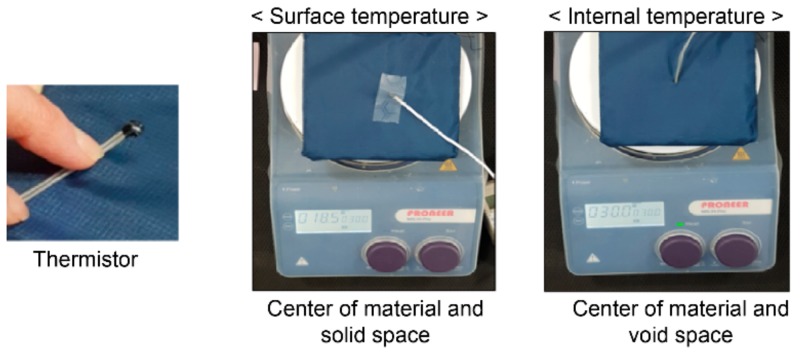
Temperature measurement of a technical material.

**Figure 5:**
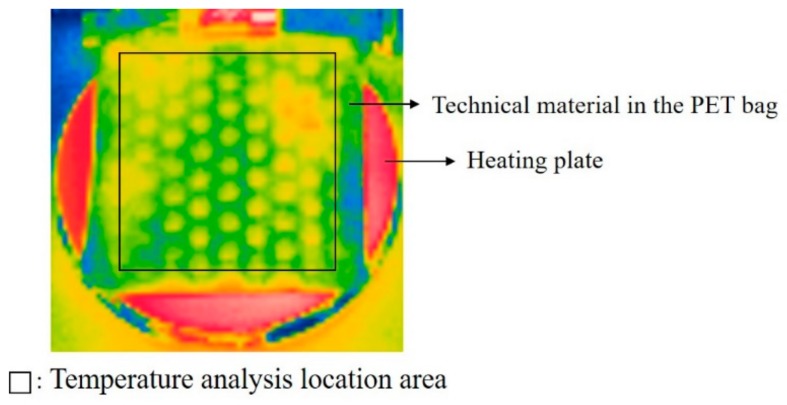
Temperature analysis location in thermogram image (top view).

The prepared samples were exposed to the standard lab environment (temperature 21 ± 2 °C, relative humidity (50 ± 5)%, wind velocity ≤0.2 m/s) for 1 h. Subsequently, the thermistor sensors were attached after being placed in polyester bags. After waiting for 10 min, the samples were moved to a climate chamber set to a winter environment (temperature 5 ± 1 °C, relative humidity RH = (60 ± 5)%, wind velocity ≤0.2 m/s). The temperature of the heating plate was set to 30 °C, which is the average temperature of skin in winter [37], and power was supplied during the experiment to maintain a constant temperature. The changes in temperature according to time were then examined with the technical materials placed on the heating plate. The surface and internal temperatures of the technical materials were measured using the Thermistor (LT-8AB, Gram, Co., Takasago, Japan) instrument, from the center of the top solid frame of the surface of the polyester bag and the void space between the legs of the technical materials with a hole bored into the center of the polyester bag, respectively. That is, the surface temperature was measured at the top surface of the technical materials, and when measuring the internal temperature, the space around the leg between the top surface and the bottom surface was measured. Here, the technical materials and sensors were not in contact to precisely measure the internal temperature (Figure 4). To examine the temperature changes in the technical materials, the temperature was measured after initially placing the materials on the heating plate of the artificial climate lab, and successively after 10, 20, and 30 min. After 30 min, the power of the heating plate was turned off and the temperature was measured 10 min later. Measurements took approximately 2 min, and data were collected over three runs of the experiment. The temperature data obtained from the experiment were processed using SPSS 24.0 to calculate the mean value and standard deviation. Simultaneously, an infrared ray imaging camera FLIR E75 (FLIR Systems Inc., Wilsonville, USA) was used to film the surface temperature distribution at the end of the temperature measurement to visually examine the surface temperature changes. Moreover, the overall surface temperature of the top of the sample was analyzed and compared to the thermistor temperature data. The domain of the overall temperature analysis is shown in Figure 5. 

## 3. Results and Discussion

### 3.1. Technical Material Surface and Internal Temperature Measurement

**Figure 6:**
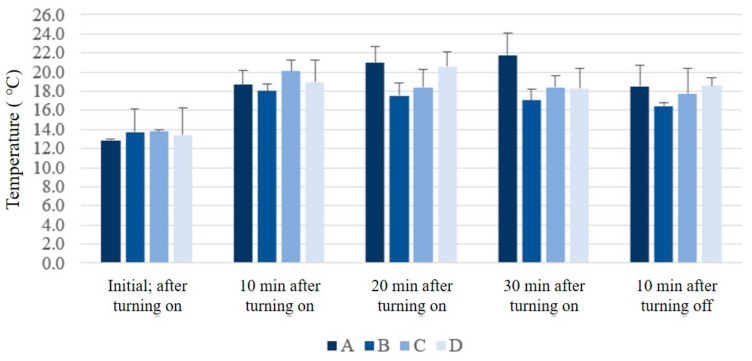
Surface temperature changes of the technical material samples according to exposure time.

The surface temperature measurement results of the technical materials are shown in Figure 6. The initial surface temperatures of the materials exposed to an artificial winter environment in a climate lab were similar. However, the temperatures of all four types of technical materials increased after placing the samples for 10 min on heating plates set to 30 °C, the average temperature of skin in winter. The gap between samples increased after 20 min, with an increase in the surface temperatures of A and D and a decrease in those of B and C. Sample A exhibited identical features to B but had a lower leg height and hence a thin air gap. Sample D had characteristics identical to those of C with a higher leg and thick air gap. Additionally, samples A and B possessed larger pore sizes than C and D owing to smaller side widths. In other words, when pore size was large (A, B), insulation was inefficient with a thin air gap because of swift thermal transmission, and when pore size was small (C, D), a thick air gap was detrimental to insulation. After 30 min, sample A had the highest surface temperature whereas B had the lowest surface temperature. Therefore, these results show that with an increase in time, insulation is superior when the pore size is large, and the air gap is wide. Meanwhile, 10 min after turning off the heating plate, samples A and D had the highest surface temperatures, whereas B had the lowest temperature. Therefore, when no power is supplied to the heating plate, samples A and D, which had higher temperatures, had better insulation owing to higher thermal capacity, and B was the most inefficient in terms of insulation.

Overall, while the heat was continuously supplied by the heating plate, sample A had a lower initial temperature, and its surface temperature rapidly and consistently increased with time. However, the surface temperature of B decreased with time and reached the lowest temperature after the heating plate was turned off. In contrast, after the first 10 min of heating, samples C and D, which had smaller pores than A and B, tended to maintain the same temperature, even after turning off the heating plate.

Sample A had relative larger pores than C and D and a lower leg height than B and D, and the results indicate that these conditions were highly inefficient for insulation. However, sample B had the same pore size as A, but B displayed efficient insulation, suggesting that thermal transmission was slow because the leg height of B was higher than that of A. According to Choi [38], procuring a fixed air gap by increasing the width and lowering the density is a method for increasing the insulation of the material, as efficient confinement of air with low thermal conductivity is crucial to insulation. It can be concluded that the presence of a thick air gap enhances insulation. In other words, the effect of air gap thickness becomes less significant when the pores are small.

After turning off the heating plate, the temperatures of most materials decreased, with the rate of this decrease highest for sample A. Samples A and D had the highest surface temperatures and B had the lowest. A high temperature decrease rate indicates that a significant amount of heat is abstracted after the heat supply is removed, implying that a high surface temperature provides superior insulation. Therefore, considering both surface temperature and temperature decrease rate after removal of the heat supply, sample D with small pore size and 1.5 cm leg height was most efficient in terms of insulation. Ziaei and Ghane [39] stated that a spacer fabric of 6.0 mm thickness had better thermal resistance than that of 3.0 mm thickness and hence better insulation, which agrees with this study. Moreover, according to Lee et al. [28], for a 3D spacer knit fabric with identical thickness, one layer with a large pore size had superior insulation than two layers with small pore sizes, implying that 3D printed materials using TPU did not depend on the pore size for insulation, leading to different thermal transmission properties compared to conventional fabrics. Additionally, when the ease allowance of clothing is increased, i.e., as length or width is added to the body measurements to expand the clothing volume, the air gap increases. However, a convection current is observed, and therefore, after a certain threshold, the insulation decreases [40]. Lee et al. [41] also stated that an air gap of more than 5 cm was detrimental to insulation. Therefore, a method that combines new materials rather than expanding the clothing air gap for enhanced insulation is worthy of consideration, as described here. 

**Figure 7:**
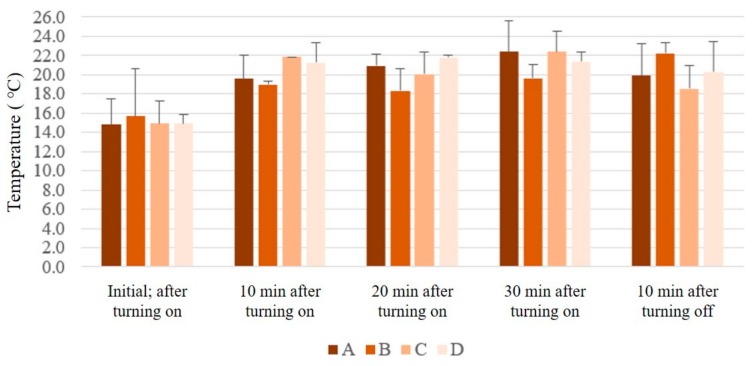
Internal temperature changes of technical material samples according to exposure time.

The internal temperature measurement results are shown in Figure 7. The initial internal temperatures were similar for all samples, and all samples displayed an increase in temperature 10 min after placing them on the heating plates. The measurements after 20 min showed an increasing trend for the internal surface temperatures of A and D, whereas the internal temperatures of B and C decreased. After 30 min, sample A displayed the highest temperature with a consistent increase, whereas B had the lowest temperature. Meanwhile, the temperatures of all samples decreased (with the exception of B) after the power was turned off. Overall, the internal temperature of sample A consistently increased when heat was supplied, whereas the temperatures for other samples increased and then decreased with prolonged time. Sample B exhibited the lowest temperatures for all measurements when heat was supplied. However, after the heating plate was turned off, sample B showed the highest temperature, and sample C exhibited the highest temperature decrease rate as well as the lowest temperature. This implies that a large pore size and thick air gap are also beneficial for the insulation of internal air temperature. In comparison to the surface temperature results, sample A had the worst insulation after the heating plate was turned on and B was the most efficient. When the heating plate was turned off, D was superior in terms of surface insulation and B was superior in terms of internal insulation. Therefore, when utilizing the developed 3D spacer materials in combination with clothing, it is disadvantageous to use structures with large pore sizes and thin air gaps, and having a large air gap regardless of pore size is expected to be most efficient. Numerous studies argue that thermal insulation increases as air gap width increases, but when the air gap is sufficiently wide, natural convection occurs and affects material insulating capabilities [18,42,43]. As a solution to this issue, wearing multi-layered clothing creates trapped air, providing the most efficient insulation [44]. In this study, the leg height was not varied. However, the efficient insulation of sample B, despite an increase in the air gap thickness to 1.5 cm, was considered to be due to the trapping of air flow by the polyester bag, and possibly delayed conduction owing to the high legs of sample structure, as well as delayed radiation because of its numerous legs. This encapsulates the human body by formulating a fixed air gap in a method distinctive to multi-layered clothing. Further studies are required for more in-depth investigations of this phenomena.

### 3.2. Overall Surface Temperature Distribution of the Technical Materials

**Figure 8:**
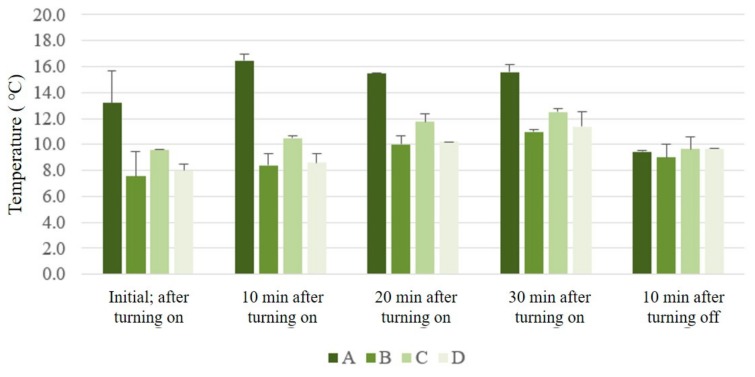
Overall temperature changes according to sample exposure time.

The thermogram images of surface temperatures of each sample are shown in Table 3. The pores of the technical materials cannot be seen in the initial thermogram image of samples B and D. This is because the air gaps in samples B and D were thicker than those in A and C, and therefore, thermal transmission was delayed. However, as seen in the latter images (Table 3), the presence of pores could be confirmed for all samples. When the heating plate was turned off, pores disappeared in these images for all samples except B. The temperature change in all samples can be observed in Figure 8. Unlike the surface and internal temperature measured using the thermistor sensor, sample A had the highest initial temperature, and B had the lowest initial temperature because of the temperature of the pores. However, for almost all measurement times, sample B had the lowest temperature, which agrees with the analyzed surface and internal temperatures and confirms the importance of a large pore size and thick air gap for efficient insulation. 

## 4. Conclusions

In this study, technical materials with 3D spacer fabric structures were designed for cold environments and were subsequently 3D printed. The surface and internal temperature data were analyzed to determine their thermal transmission properties.

The results show that the heat transfer characteristics of technical materials were different, even though they were made from the same TPU, because of their different thicknesses and pore sizes. The developed technical materials have superior insulation when pore size is large and the air gap is thick in the case where heat is supplied from the heat source. However, when the heat supply is removed, a thick air gap becomes a more dominant factor for insulation, regardless of the pore size. Generally, when people work in a cold outdoor environment, the body is subjected to heat loss. Therefore, supplements must be utilized to maintain the balance of metabolic processes by preventing heat loss. If technical materials are inserted inside clothing to procure an air gap for this purpose, enlarging the air gap is more advantageous than enlarging the pore size. 

In general, heat transfer is a very important factor related to the comfort of clothing. There has been recent increasing utilization of fabrics formed by weaving composite fabrics for use in various industries and attempts to combine 3D polymer filaments with textile fabrics [45]. In applying these new materials, not only physical but also thermal property analyses are meaningful. In this study, a heating plate can be used to approximate a human body that is emitting heat at body temperature. In cold outside environments, the body regulates its temperature to maintain homeostasis, and heat is transferred to the fabric surface. However, the results of this study show that if there is a technical material used on the human body, the heat flow efficiency depends on its leg height and surface area. Therefore, it was found that it is efficient to properly adjust the three-dimensional shape of technical materials according to environmental conditions and human activity.

Anyone can now access the tool of 3D design and printing, and the materials that may be used in this process are increasing day by day, which has the great advantage of easy access for designers. Through this challenging process of trial and error, optimal design, together with techniques for applying new materials to clothing, is considered possible. However, if the developed technical material is combined with clothing, the effect of this material on the comfort of clothing will differ depending on the structure of the technical material. In addition, 3D printed materials made from TPU afford cushioning owing to the original properties and thickness of the TPU; thus, such materials can protect the body from various substances in the work environment.

Based on the results of this study, fundamental data regarding the structural thermal properties of the developed technical materials can be assembled. This research is also significant because the fabric has been developed from the point of view of exploiting the latest 3D printing technology, which is subject to increasing popularity and research interest. However, this experiment tested only the technical materials, and their insulating capabilities within real clothing still need to be verified. Additionally, in view of various working conditions, their thermal transmission properties should be analyzed in terms of wind velocity, humidity, and intensity of labor.

In future, the applicability of the suggested 3D technical materials should be investigated by analyzing the thermal properties of the technical materials when grafted to functional clothing. Furthermore, variables such as the amount of clothing and clothing layers must be considered to predict the insulation of technical materials. 

## Figures and Tables

**Table 1:** Nomenclature for the technical material design.

Material Design	Side Width (Unit: cm)	Leg Height (Unit: cm)	Pore Size (Unit: cm)
A	0.2	0.5	0.9
B	0.2	1.5	0.9
C	0.5	0.5	0.6
D	0.5	1.5	0.6

**Table 2:** Product specifications of thermoplastic polyurethane (TPU).

Specifications of TPU	
Print temperature	210–235 °C
Baseplate temperature	50–75 °C
Heat deflection temperature	85–110 °C
Melt flow index	14–28 g/10 min
Tensile yield strength	21–36 MPa
Elongation at break	26–60%
Flexural strength	60–97 MPa
Flexural modulus	1.8–3.0 G/Pa
Impact strength	120 kJ/m^2^
Filament diameter	1.75 mm (±0.05 mm)

**Table 3:** Thermogram images of the material samples.

**Heating Plate Condition**	**A**	**B**	**C**	**D**	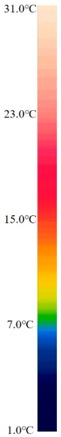
Initial; after turning on	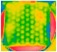	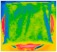	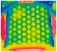	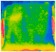	
10 min after turning on	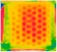	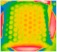	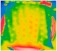	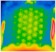	
20 min after turning on	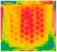	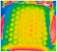	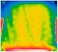	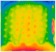	
30 min after turning on	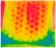	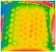	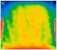	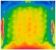	
10 min after turning off	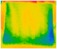	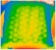	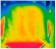	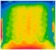	
